# 3D‐printed autoclavable plant holders to facilitate large‐scale protein production in plants

**DOI:** 10.1002/elsc.202200001

**Published:** 2022-08-15

**Authors:** Ling Chuang, Anton Enders, Sascha Offermann, Janina Bahnemann, Jakob Franke

**Affiliations:** ^1^ Centre of Biomolecular Drug Research Leibniz University Hannover Hannover Germany; ^2^ Institute of Technical Chemistry Leibniz University Hannover Hannover Germany; ^3^ Institute of Botany Leibniz University Hannover Hannover Germany; ^4^ Institute of Physics University of Augsburg Augsburg Germany

**Keywords:** additive manufacturing, agroinfiltration, autoclavable 3D printing material, *Nicotiana benthamiana*, vacuum infiltration

## Abstract

The Australian tobacco plant *Nicotiana benthamiana* is becoming increasingly popular as a platform for protein production and metabolic engineering. In this system, gene expression is achieved transiently by infiltrating *N. benthamiana* plants with suspensions of *Agrobacterium tumefaciens* carrying vectors with the target genes. To infiltrate larger numbers of plants, vacuum infiltration is the most efficient approach known, which is already used on industrial scale. Current laboratory‐scale solutions for vacuum infiltration, however, either require expensive custom‐tailored equipment or produce large amounts of biologically contaminated waste. To overcome these problems and lower the burden to establish vacuum infiltration in new laboratories, we present here 3D‐printed plant holders for vacuum infiltration. We demonstrate that our plant holders are simple to use and enable a throughput of around 40 plants per hour. In addition, our 3D‐printed plant holders are made from autoclavable material, which tolerate at least 12 autoclave cycles, helping to limit the production of contaminated waste and thus contributing to increased sustainability in research. In conclusion, our plant holders provide a simple, robust, safe and transparent platform for laboratory‐scale vacuum infiltration that can be readily adopted by new laboratories interested in protein and metabolite production in *Nicotiana benthamiana*.

Practical application

Transient expression in *Nicotiana benthamiana* provides a popular and rapid system for producing proteins in a plant host. To infiltrate larger numbers of plants (typically >20), vacuum infiltration is the method of choice. However, no system has been described so far which is robust to use and can be used without expensive and complex equipment. Our autoclavable 3D‐printed plant holders presented here will greatly reduce the efforts required to adopt the vacuum infiltration technique in new laboratories. They are easy to use and can be autoclaved at least 12 times, which contributes to waste reduction and sustainability in research laboratories. We anticipate that the 3D printing design provided here will drastically lower the bar for new groups to employ vacuum infiltration for producing proteins and metabolites in *Nicotiana benthamiana*.

**Abbreviations**
CFU: colony forming units
GFP: green fluorescent protein
*N. benthamiana*
: *Nicotiana benthamiana*
PLA: polylactic acid

## 1 INTRODUCTION

In recent years, the Australian tobacco plant *Nicotiana benthamiana* has become an important host for protein production and for metabolic engineering, with wide use from laboratory scale up to industrial scale [1, 2, 3, 4, 5, 6, 7, 8]. This process is also called transient expression, because it is not necessary to create stable transgenic plants; instead, fully expanded leaves of *N. benthamiana* plants are infiltrated with agrobacteria suspensions which transfer the gene sequences to be expressed. *N. benthamiana* is an attractive host because it is capable of introducing typical eukaryotic post‐translational modifications, contaminations with animal and human pathogens are unlikely, and the process is highly scalable. Two main techniques are commonly used for agroinfiltration, namely manual infiltration via needle‐less syringes, and vacuum infiltration. Because vacuum infiltration is much faster than syringe‐infiltration, all large‐scale and industrial applications are based on this approach. Even though vacuum infiltration is a powerful method for high‐throughput agroinfiltration and sophisticated devices are known for industrial‐scale vacuum infiltration [9, 10, 11, 12, 13], current laboratory‐scale vacuum infiltration methods possess severe disadvantages for groups entering the field. So far, only three reports describe their setup for vacuum infiltration in a research laboratory environment in detail [14, 15, 16]. The system based on a modified freeze‐dryer from the Osbourn group [3, 14] provides a powerful platform for simultaneous infiltration of four plants, but their design of custom‐made plant holders is not publicly available and requires considerable engineering skills, hampering transfer of this technique to new labs. Leuzinger et al. (2003) employ a modified desiccator plate [15, 17], which limits the throughput to a single plant per infiltration cycle. Lastly, Loh and Wayah (2014) wrap their plants in aluminum foil as support [16], which slows down the vacuum infiltration process and results in large quantities of contaminated waste. In addition, other groups have used custom‐made plant holders or frameworks, but did not report technical details [18, 19, 20, 21, 22, 23, 24]. From hydroponics, pot lids are known which could be used “off‐label” as plant holders for vacuum infiltration; however, they are not designed for quick attachment and detachment, and possess sharp edges (Supplementary Figure S1). In this technical report, we describe 3D‐printed plant holders for vacuum infiltration. Our plant holders are easy and quick to apply, customizable, affordable, and are made of autoclavable material that tolerate at least 12 autoclave cycles. The setup that we describe here enables infiltration of around 40 plants per hour, which is suitable to conduct most common experiments in a timely manner. By sharing our 3D model, our plant holders can easily be reprinted by others. Our system therefore considerably lowers the barrier to use vacuum infiltration in new laboratories and will therefore be an important step to disseminate this platform as an important tool for protein production and metabolic engineering in a plant host.

## 2 MATERIALS AND METHODS

### 2.1 Bacterial strains, plasmids, and plant material

The vector pEAQ‐GFP‐*HT* for expression of the green fluorescent protein (GFP) gene in plants was previously described by Sainsbury et al. [25] *Nicotiana benthamiana* LAB strain [1] was grown from seeds in a greenhouse with 11 h illumination per day, at a temperature between 21°C and 23°C, and humidity between 60% and 85%. Natural light was supplemented with artificial light from 60 klm growth lamps whenever sunlight was lower than 60 klux. Plants were grown in 9 cm pots. The soil contained 70% organic material from moderately decomposed bog peat, white peat and sod peat. The rest is composed of clay, calcium carbonate and NPK fertilizer (340 mg/L N, 380 mg/L P_2_O_2_, 450 mg/L K_2_O, 130 mg/L S, 160 mg/L Mg). Details of common greenhouse conditions are also described in [14].

### 2.2 3D‐printing the plant holders

The plant holders were designed in Solidworks 2020 (Dassault Systèmes, Vélizy‐Villacoublay, France). First prototypes for initial optimisation were 3D‐printed using polylactic acid (PLA) material and the PRUSA Mini+ (Prusa Research, Prague, Czech Republic) Fused Filament Fabrication 3D printer. For final experiments, the plant holders were 3D‐printed using the ProJet 2500 plus MultiJet 3D printer (3D Systems, Rock Hill, SC, USA). The printing material used was M2S‐HT90 (3D Systems), which forms solid polyacrylate after being exposed to UV rays during the printing process. The support material used was M2‐SUP (3D Systems). After completion of the print, the parts were cooled at –18°C in a freezer for 10 min, and then separated from the print platform. The printed parts were then placed in a hot water vapor bath to melt the wax‐like support material for 20 min. Afterwards, the parts were placed in a heated oil bath (paraffin oil at 65°C) to dissolve the support material adhering to the printed model surface and submerged in an ultrasonic bath with paraffin oil in order to dissolve any residual support material. Finally, oil residues were removed using soap water at 65°C (Fairy Ultra detergent, Procter & Gamble, Cincinnati, OH, USA) in another ultrasonic bath.

### 2.3 Vacuum infiltration and GFP expression

*Nicotiana benthamiana* plants grown in a greenhouse for 4–6 weeks were used for the vacuum infiltration. 4‐ and 5‐week‐old plants typically reach a maximum diameter of 15 and 20 cm, respectively, in our growth conditions, suitable for 400 mL beakers. Six‐week‐old plants might require 600 mL beakers. Vacuum infiltration was carried out in a 9.2 L ROTILABO desiccator (Carl Roth, Karlsruhe, Germany), which can accommodate 3–4 *N. benthamiana* plants for simultaneous infiltration, depending on the size of plants and beakers used. Vacuum was applied using the membrane pump MZ 2 NT (Vacuubrand, Wertheim, Germany) equipped with a manometer to verify that the desiccator is correctly sealed. Please note that oil‐based pumps are not suitable for agroinfiltration as the water vapor generated at lower pressures will condense in the oil (unless a cooling trap is used).

**FIGURE 1:**
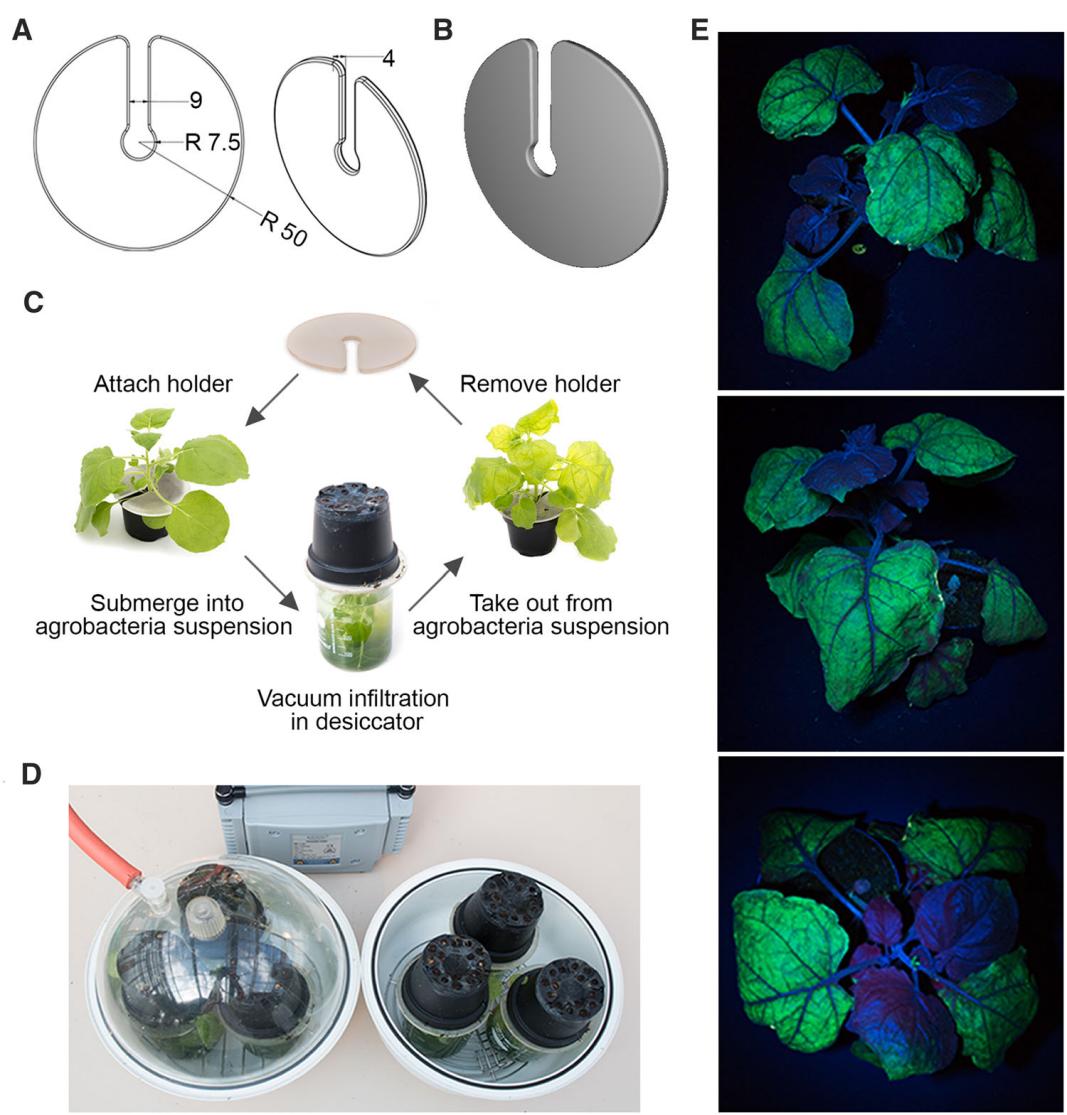
Plant holder design. (A) Shows the schematic drawing of the holder. Dimensions are provided in millimetres. All edges were rounded (fillet) with a 1 mm radius to avoid damage to the plants. (B) Shows the top view of an actual holder. (C) Shows a schematic representation of the vacuum infiltration cycle using the plant holder. (D) Shows the complete vacuum infiltration setup; by alternating between two desiccators, around 40 plants can be infiltrated per hour. (E) Successful vacuum infiltration of *Nicotiana benthamiana* as demonstrated by transient *GFP* expression. A representative plant is shown from multiple angles. Plants were infiltrated with *Agrobacterium tumefaciens* containing the pEAQ‐GFP‐*HT* plasmid [25]

For GFP expression, suspensions of *Agrobacterium tumefaciens* containing the plasmid pEAQ‐GFP‐*HT* were provided in three 400 mL glass beakers (VWR, Darmstadt, Germany; article number 213–0479, external ø: 80 mm, height: 110 mm) and placed on a stainless‐steel stand (microwave and steamer grid rack, ø: 20 cm, feet height: 4 cm, flipped to use) in the desiccator; whenever required, the beakers were topped up to keep the fill level close to the beak. The plant holders were put over the plant stem at the stem base, and the inverted plants were placed into the beakers filled with *A. tumefaciens* suspensions. Infiltration was carried out for 3 min at maximum pump power; the final pressure of ca. 30 mbar was reached after 1.5 min and held for another 1.5 min, resulting in 3 min vacuum cycles. The overall setup, which includes alternating between two desiccators for maximum throughput, is shown in Figure 1D. After infiltration, plants were kept in the greenhouse for 7 days at 21°C–23°C, 11 h/13 h light/dark photoperiod, and 60%–85% humidity before visualisation.

### 2.4 Visualisation of GFP expression

GFP‐infiltrated plants were placed inside a photo box (60 × 60 × 60 cm; HAVOX, Vendome, France) and illuminated with a Xite RB‐GO (Royal Blue, Green Only; excitation 440–460 nm) fluorescence flashlight (NIGHTSEA, Lexington, MA, USA). Pictures were taken with a Canon 70D DSLR camera equipped with a combination of Y2 Pro (Yellow) and X1 (Green) filters (Hoya, Tokyo, Japan) to filter out background blue light and red chlorophyll autofluorescence, respectively.

### 2.5 Side‐by‐side comparison of plant holders

Two setups from the literature [16, 18] for lab scale vacuum infiltration were reproduced to compare their operation times to our holders. For the method from Loh and Wayah, pots were wrapped with two pieces of aluminum foil and fixated in the beaker using two spatulas [16]. For the method employing hand‐made aluminum foil holders shown in a review by Lomonossoff and D'Aoust [18], holders were assembled from six layers of aluminum foil and folded in a proper shape without exposing sharp edges. Seven participants, four experienced and three new users, were asked to repeat the following actions for four times using each holding system for three plants: taking out three plants with their holding items from beakers with water in the desiccator; disassembling the holding setup; and re‐assembling the holding items onto three new plants. The operation time was recorded in seconds and plotted with R.

### 2.6 Determination of colony forming unit counts

Each plant holder was soaked in 30 mL infiltration suspension of *Agrobacterium tumefaciens* containing pEAQ‐GFP‐*HT* at OD_600_ 0.6 in a plastic bag for 1 h. The holder was then cleaned using three different ways (wiping with tissue, wiping with 70% ethanol, and autoclaving) and then soaked in 10 mL of lysogeny broth (LB) medium containing 50 μg/mL kanamycin for 1 h at 20°C in a plastic bag. The LB media with bacteria were then transferred to 50 mL falcon tubes and harvested by centrifugation at 5000 × g for 15 min. The harvested bacteria were then resuspended in 200 μL of LB medium. Resuspended bacteria were serially diluted and plated on LB agarose plates containing 50 μg/mL kanamycin. Colony numbers were counted on the plates with 10^5^ and 10^6^ dilution factors for calculating colony forming units (CFU) per holder. Three holders per cleaning treatment were analyzed; the full experiment was carried out three times independently.

### 2.7 Autoclaving plant holders

The holders were autoclaved at 121°C for 15 min in a common laboratory autoclave.

## 3 RESULTS AND DISCUSSION

### 3.1 Optimisation of plant holder design

Our first task was to develop an optimal design for the 3D‐printed plant holders for vacuum infiltration. To save material costs during this initial optimisation, plant holders were printed using PLA at this stage. The following criteria were considered as critical: A sufficiently large gap for rapid passage of air; no sharp edges to prevent damage to the plants; ease of use; tolerance towards a wide range of plant sizes, particularly regarding the stem diameter; as little material as possible to save costs, but as much material as required for sufficient stability. After multiple design‐build‐test‐learn cycles, we obtained the design shown in Figure 1A and 1B which was considered as optimal for our vacuum infiltration setup. An example showing transient expression of the green fluorescent protein (GFP) gene is shown in Figure 1E.

### 3.2 Vacuum infiltration and side‐by‐side comparison

After optimizing the design of the plant holders, we next wanted to see how efficient our system would work for infiltrating a larger number of plants. The time requirement for infiltrating a large number of plants by vacuum is determined by three factors: (1) How many plants can be infiltrated at the same time; (2) how long one infiltration cycle takes; (3) how much time is needed in between cycles to remove the infiltrated plants and set up the next plants for the infiltration. While factors 1 and 2 depend on the size of the vacuum chamber in comparison to the plant sizes and the pump used, respectively, factor 3 was considered as central for evaluating the performance of our 3D‐printed plant holders. We therefore measured the operation times between infiltration cycles during multiple rounds of infiltration. Using two desiccators alternatingly as shown in Figure 1C and 1D, it normally required 1–2 min to operate the following actions: Taking out the three infiltrated plants from the desiccator, removing the holders, installing the holders onto three new plants, submerging the plants in bacteria suspension and placing them in the desiccator. This preparation for the next cycle can be completed within the 3 min vacuum time of the other desiccator. Switching off the pump, releasing the vacuum, transferring the lid to the other desiccator, and restarting the pump took around 60 s, which add to each infiltration cycle. Taking these times together, we obtained consistent cycle times of around 4 min per infiltration cycle. As three plants are infiltrated per cycle, we achieved robust infiltration rates of around 40 plants per hour. In our experience, a scale of 50–100 plants per infiltration batch is sufficient for most applications in research laboratories and can be handled with common downstream processing equipment.

**FIGURE 2:**
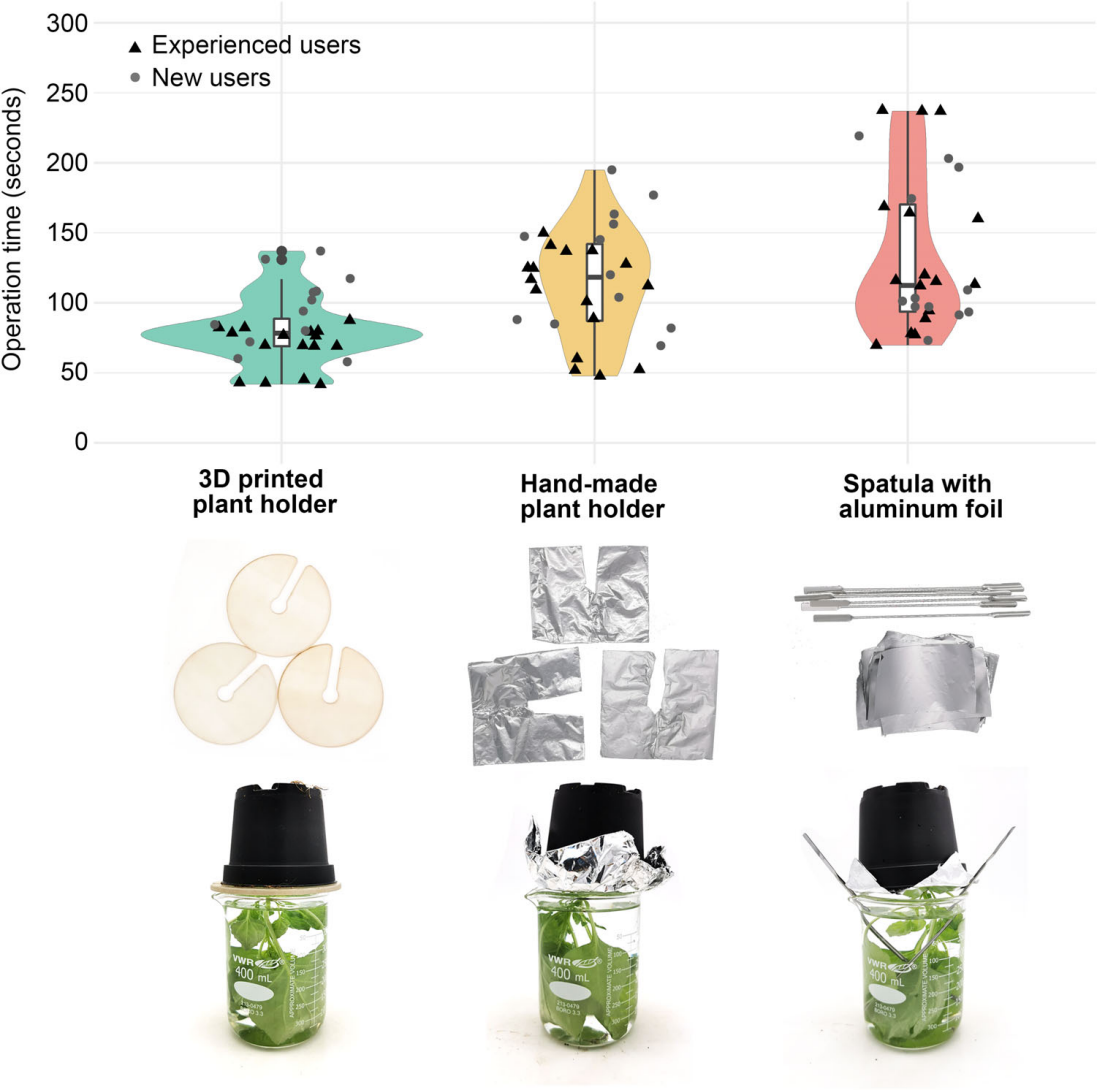
Side‐by‐side comparison of operation times of 3D‐printed holders compared to two previously described vacuum infiltration systems [16, 18]. The violin plot shows the operation times in seconds for seven participants to complete the following actions, which simulate a real vacuum infiltration cycle: taking out three plants with their holding systems from the desiccator; disassembling the holding system; re‐assembling the holding system to three new plants. Each of the seven participants repeated the actions four times. Among the participants, four were experienced users (black triangles), and three were new to vacuum infiltration (gray circles). Below the violin plot, holding items as well as holding assemblies are shown

To confirm that the system described here performs better in terms of speed, ease of use and reliability, we performed a side‐by‐side comparison with two other setups based on previous reports, one based on hand‐made aluminum foil holders [18], the other based on spatulas as support (Figure 2) [16]. These two systems were selected because they are the only reported methods that can be reproduced with common laboratory equipment without custom‐made equipment. The experiment included a panel of seven participants, four of which had previous experience with vacuum infiltration and three were new users. Our results show that using our 3D‐printed plant holders is faster (mean values; experienced users: 68 vs. 105 and 137 s; new users: 96 vs. 128 and 130 s, respectively), and more consistent (standard deviation; experienced users: 16 vs. 35 and 58 s; new users: 26 vs. 42 and 52 s, respectively). Also, our 3D‐printed plant holders could be re‐used easily, whereas the other methods showed limited long‐term stability and led to the accumulation of biologically contaminated aluminum foil waste. Also, sharp edges from aluminum foil easily caused plant injury. While we observed a noticeable training effect for our 3D‐printed plant holders, even unexperienced users achieved operation times fast enough for maintaining the overall infiltration cycle time of ca. 4 min per cycle. We conclude that our 3D‐printed plant holders and the setup described here greatly facilitate access to laboratory‐scale vacuum infiltration technology to other laboratories.

### 3.3 Autoclavable material for increased biosafety and reduced waste production

During the vacuum infiltration process, the plant holders become contaminated with transgenic *Agrobacterium tumefaciens* strains. To avoid cross‐contaminations and to comply with biosafety regulations, it is therefore mandatory to disinfect the plant holders after use. At the same time, it is highly desirable to re‐use the plant holders to limit the waste produced. We therefore aimed to produce our plant holders by 3D‐printing of autoclavable material, namely M2S‐HT90 [26, 27]. The material has a heat deflection temperature of 90°C and is capable of meeting USP Class VI standards for biocompatibility.

**FIGURE 3:**
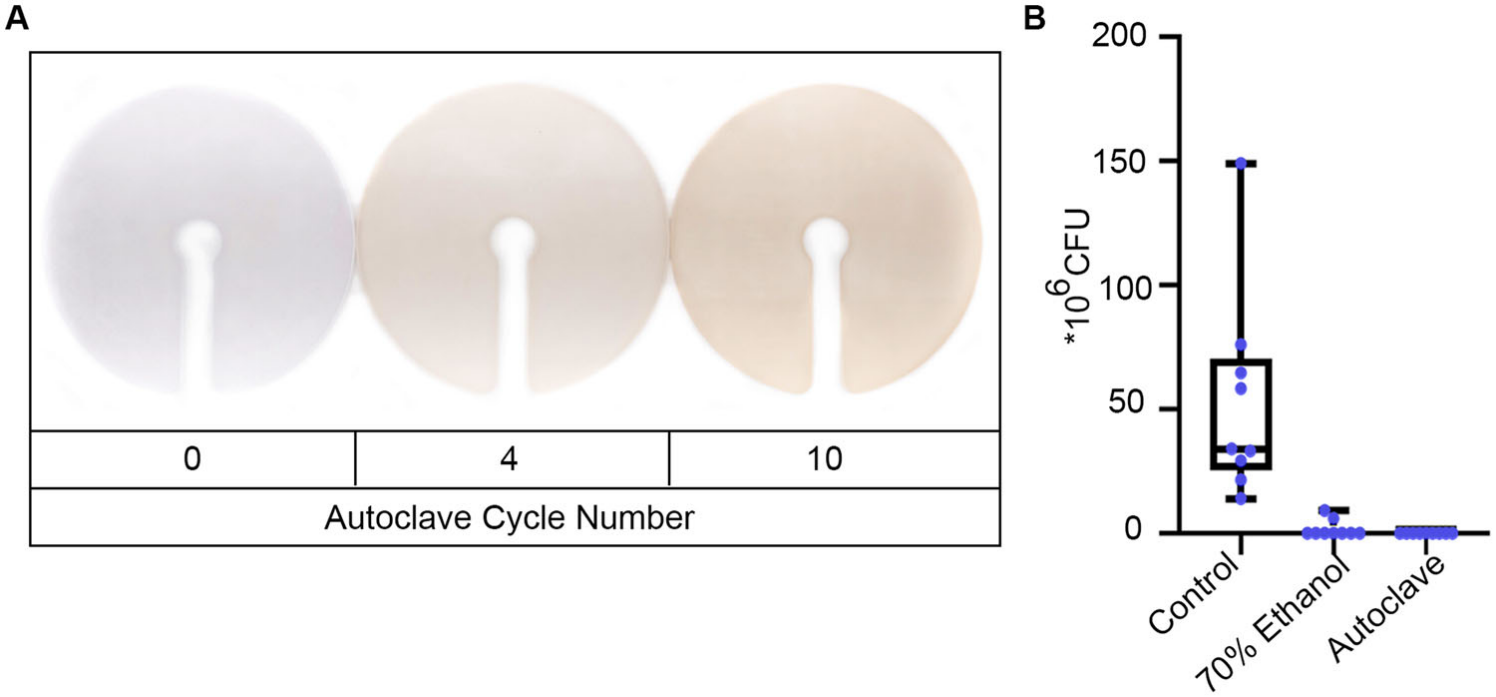
Plant holders described here can be autoclaved for disinfection. (A) Plant holders showed a gradual darkening over multiple autoclave cycles, but no other deterioration. (B) Autoclaving is a highly effective method to sterilise plant holders. All plant holders used for the sterilisation experiment had already been autoclaved four times, demonstrating that they tolerate multiple autoclave cycles. Abbreviation: CFU, colony forming units

To confirm that plant holders printed from M2S‐HT90 are compatible with autoclaving, we subjected these holders to a total of 12 autoclave cycles. Although we noted a gradual darkening of the holders (Figure 3A), no other deteriorations such as cracks or deformations were noticeable, confirming that our plant holders are compatible with autoclaving and can be re‐used multiple times. In comparison, pot lids from hydroponics exhibited a clear deformation already after four autoclave cycles (Supplementary Figure S2).

Next, we wanted to confirm that autoclaving the plant holders is an efficient way to disinfect the plant holders, to ensure that potential microscopic cracks in the surface structure of the plant holders do not provide protection for the bacterial cells during autoclaving. We therefore conducted an experiment to quantify the bacteria on the surface of contaminated holders after different disinfection treatments. Plant holders were first soaked in a typical *Agrobacterium tumefaciens* suspension (OD_600_ 0.6) in a plastic bag for 1 h. The plant holders were then cleaned in three different ways: (1) Wiping the surface with tissue paper (control), (2) wiping the surface with 70% ethanol, and (3) autoclaving the holder in a plastic bag. The remaining bacteria on the holder surface were then collected in LB medium, serially diluted and plated for calculating CFU per holder. From our results (Figure 3B), we found that autoclaving is highly effective to remove all bacteria from the re‐used holder surface, while 70% ethanol wipe was not consistently effective. As we used holders which had been autoclaved already four times for this experiment, our data also demonstrates that the holders retain full performance even after multiple autoclave cycles. These autoclavable plant holders thereby help to reduce waste and comply with biosafety regulations.

Based on the material usage estimation in the printer software, one autoclavable plant holder costs ca. 20€ when made from the material M2S‐HT90 with our MultiJet Printing (MJP) printer. With a more common fused deposition modelling (FDM) printer and cheaper materials such as PLA or heat‐resistant polycarbonate (PC) blend, lower costs of approximately 1–2€ per holder could be achieved, but the autoclave stability might be impaired. Laboratories without access to a 3D printer can also employ commercial print services, which offer heat‐resistant materials such as polyamide (PA) PA2200 at costs around 25–30€ per holder. Even though no costs for other vacuum infiltration systems have been published, we assume that our 3D‐printed plant holders fill the gap between simple home‐made plant holders [15, 16, 17, 18] and more complex proprietary plant holders [9, 10, 11, 12, 13], and hence form an ideal compromise between ease‐of‐use and financial investment.

## 4 CONCLUDING REMARKS

We report here the optimisation and evaluation of autoclavable 3D‐printed plant holders for vacuum infiltration of *Nicotiana benthamiana* for protein production. We demonstrate that these plant holders permit consistent infiltration rates of around 40 plants per hour with the setup described here. The 3D‐printed plant holders can be autoclaved at least 12 times without reducing their performance, helping to limit waste and facilitate biosafety measures. We are aware of commercial pot lids from hydroponics. However, our own approach offers the additional feature to adapt the 3D printing design to individual needs. Furthermore, the observed long‐time stability testing 12‐fold autoclaving outcompeted the stability of commercial products. Taken together, the autoclavable 3D‐printed plant holders described here will enable other laboratories to quickly adopt vacuum infiltration for protein production and metabolic engineering in *N. benthamiana* using a robust, easy‐to‐use, and re‐usable system.

## CONFLICTS OF INTEREST

The authors have declared no conflicts of interest. The CAD templates of the plant holder (*.STL and *.SLDPRT formats) are available in the supplementary material of this article.

## Supporting information

SUPPORTING INFORMATION
