# Supplementary material for: 3D‐printed autoclavable plant holders to facilitate large‐scale protein production in plants
Source: Eng Life Sci. 2022 Aug 15;22(12):803–10. doi: 10.1002/elsc.202200001 (PMC9731595; doi:10.1002/elsc.202200001)
Supplement: Supplementary file 1 — SUPPORTING INFORMATION [file ELSC-22-803-s001.pdf]

Supporting Information

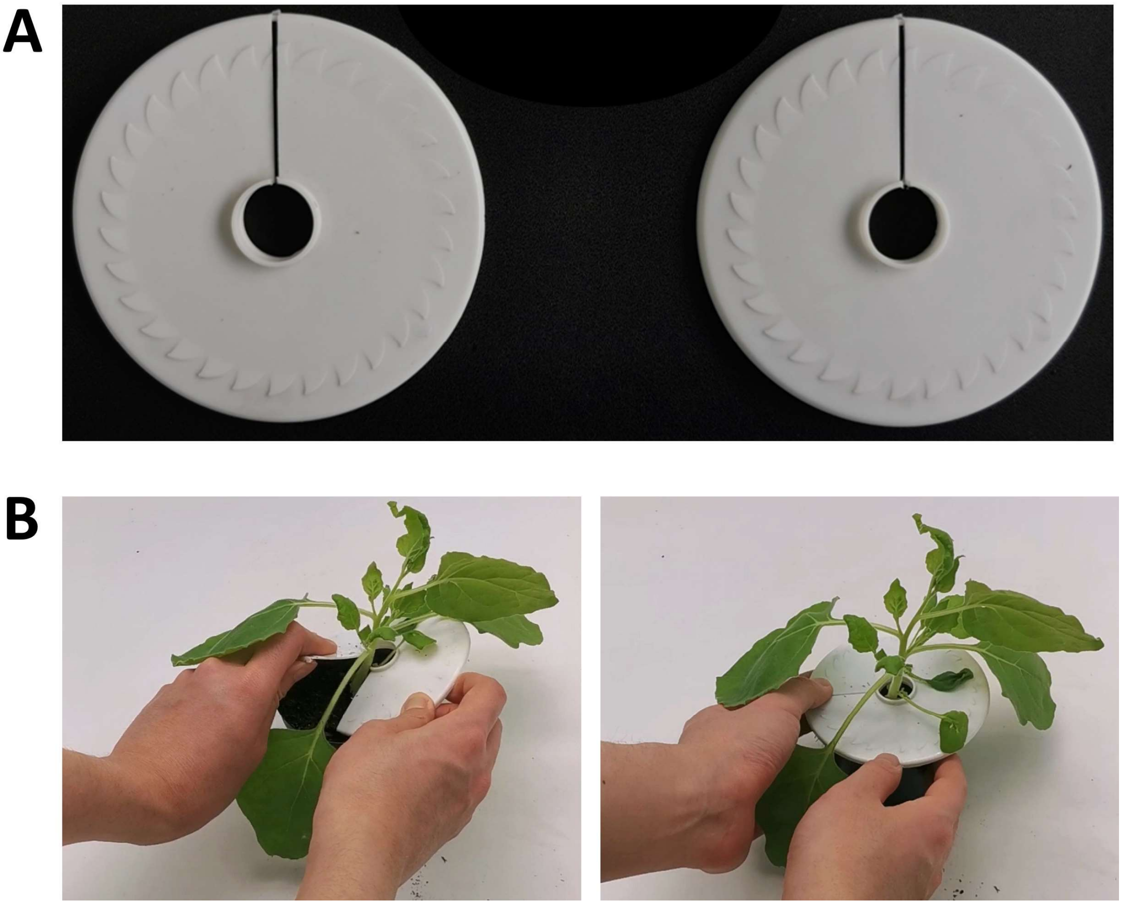

Supplementary Figure 1. Pot lids from hydroponics could serve as plant holders for vacuum infiltration, but are not designed for quick attachment and detachment, and contain sharp edges which can easily cause injury of plant stem or leaves. **A.** Pot lids obtained from Greenhope (Germany; article number 26317) and ServoVendi (Spain, article number 8014) with identical appearance. **B.** Process of attaching the hydroponics pot lids to *N. benthamiana* plants for vacuum infiltration.

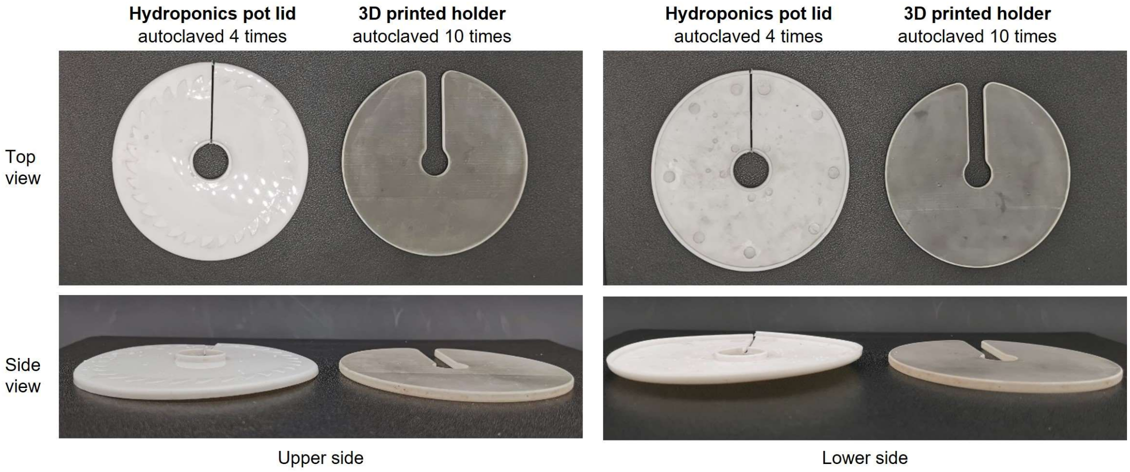

Supplementary Figure 2. Pot lids from hydroponics exhibit limited thermal stability during autoclaving compared to our 3D-printed plant holders.
